# Lamellarin O, a Pyrrole Alkaloid from an Australian Marine Sponge, *Ianthella* sp., Reverses BCRP Mediated Drug Resistance in Cancer Cells

**DOI:** 10.3390/md12073818

**Published:** 2014-06-27

**Authors:** Xiao-Cong Huang, Xue Xiao, Yun-Kai Zhang, Tanaji T. Talele, Angela A. Salim, Zhe-Sheng Chen, Robert J. Capon

**Affiliations:** 1 Division of Chemistry and Structural Biology, Institute for Molecular Bioscience, The University of Queensland, Brisbane QLD 4072, Australia; E-Mails: xiaocong.huang@uqconnect.edu.au (X.-C.H.); xue.xiao@imb.uq.edu.au (X.X.); a.salim@imb.uq.edu.au (A.A.S.); 2 Department of Pharmaceutical Sciences, College of Pharmacy and Health Sciences, St. John’s University, Queens, NY 11439, USA; E-Mails: helloyunyun@live.com (Y.-K.Z.); talelet@stjohns.edu (T.T.T.)

**Keywords:** ABC transporter, multidrug resistance, cancer, P-glycoprotein, BCRP, MRP1, lamellarin O, marine natural products

## Abstract

ATP binding cassette (ABC) transporters, such as P-gp, BCRP and MRP1, can increase efflux of clinical chemotherapeutic agents and lead to multi-drug resistance (MDR) in cancer cells. While the discovery and development of clinically useful inhibitors has proved elusive to date, this molecular target nevertheless remains a promising strategy for addressing and potentially overcoming MDR. In a search for new classes of inhibitor, we used fluorescent accumulation and efflux assays supported by cell flow cytometry and MDR reversal assays, against a panel of sensitive and MDR human cancer cell lines, to evaluate the marine sponge co-metabolites **1**–**12** as inhibitors of P-gp, BCRP or MRP1 initiated MDR. These studies identified and characterized lamellarin O (**11**) as a selective inhibitor of BCRP mediated drug efflux. A structure–activity relationship analysis inclusive of the natural products **1**–**12** and the synthetic analogues **13**–**19**, supported by *in silico* docking studies, revealed key structural requirements for the lamellarin O (**11**) BCRP inhibitory pharmacophore.

## 1. Introduction

Cancer is the leading cause of death and represents a large diverse group of diseases, of which some can spread to other sites of the body and result in systemic metastasis. The only effective therapy for metastasis is chemotherapy, which all too frequently fails due to innate or acquired multi-drug resistance (MDR). Historically, the over-expression of three ATP binding cassette (ABC) transporters, P-glycoprotein (P-gp) [1,2], breast cancer resistant protein (BCRP) [3] and/or multi-drug resistance protein 1 (MRP1) [4], have been correlated with many occurrences of MDR. Over-expression of these proteins in cancer cells can lead to premature efflux of chemotherapeutic agents, compromising treatments. The past three decades have seen numerous efforts to overcome MDR caused by P-gp, BCRP and MRP1, with direct inhibition believed to be the most promising solution. Unfortunately, although three generations of P-gp inhibitors have been developed [5], none have proved to be clinically useful. Despite these disappointing results, the need to discover clinically effective inhibitors remains as compelling as ever [6], with marine natural products representing an attractive source of bioactive chemical diversity.

The search for marine natural products as inhibitors of ABC transporters commenced some decades ago, and includes a 1992 report by Prinsep *et al.* [7] on tolyporphin from the blue-green alga *Tolypothrix nodosa* Bharadwaja, which increased the cytotoxicity of doxorubicin and vinblastine in P-gp overexpressing SK-VLB cells [7]. Following this discovery, at least a dozen classes of marine metabolites have been reported with P-gp, BCRP or MRP1 inhibitory activity from sponges [8,9], bryozoans [10], gorgonians [11], ascidians [12], sea pens [13], antinomycetes [14] as well as algae [15] and tunicates [16].

**Figure 1:**
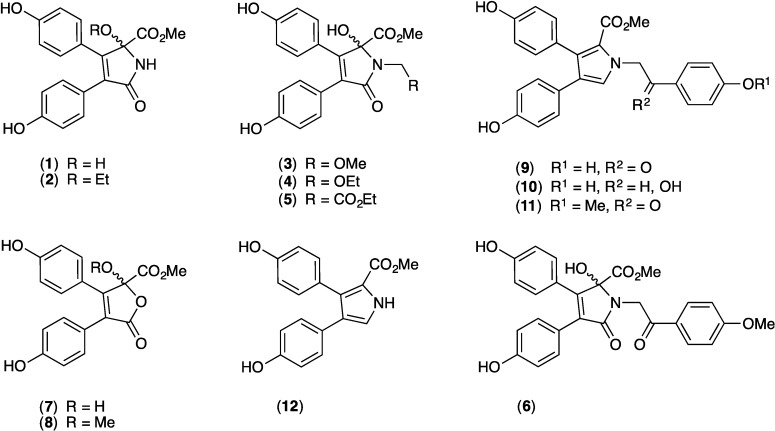
Metabolites isolated from *Ianthella* sp. (CMB-01245).

Our prior investigations into metabolites of southern Australian and Antarctic marine invertebrates, algae and microbes, have led to the discovery of a number of very promising P-gp inhibitor scaffolds, including diketopiperazines from the marine-sediment derived actinomycete *Nocardiopsis* sp. (CMB-M0232) [17], alkaloids from tunicates of the genus *Didemnum* [18], and bromoterpenes from the red alga *Laurencia filiformis* [19]. This report describes our evaluation of the ABC transporter inhibitory properties of a suite of alkaloids (**1**–**12**, Figure 1) isolated from a southern Australian marine sponge, *Ianthella* sp. (CMB-01245).

## 2. Results and Discussion

### 2.1. Cytotoxicity of **1**–**12** against SW620 and SW620 Ad300

Prior to investigating the interaction between **1**–**12** and P-gp we assessed cytotoxicity against SW620 and the MDR (P-gp over-expressing) daughter SW620 Ad300 cell line, to establish the non-cytotoxic concentration required for such studies. This study demonstrated that ianthellidones **1**–**8** and lamellarins **9**–**10** and **12** were non-cytotoxic towards SW620 and SW620 Ad300 (IC_50_ > 30 μM), while lamellarin O (**11**) exhibited comparable and moderate cytotoxicity towards both SW620 (IC_50_ 22.0 μM) and SW620 Ad300 (IC_50_ 22.3 μM) (Supplementary Table S1), with the maximal concentration for >80% survival of SW620 and SW620 Ad300 cells being 15 μM.

### 2.2. Lamellarin O (**11**) as a P-gp Inhibitor in SW620 Ad300 Cancer Cells (Calcein AM Assay)

**Figure 2:**
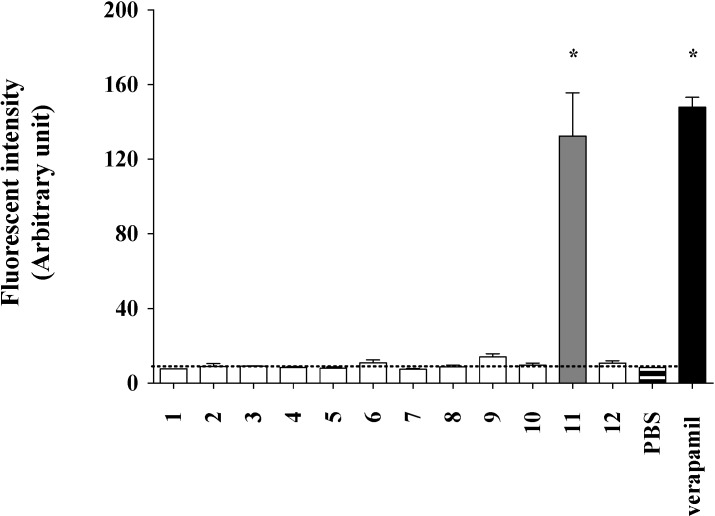
Effect of **1**–**12** on the accumulation of calcein AM. SW620 Ad300 cells in a 96-well micro-titer plate (5 × 10^4^ per well) were cultured at 37 °C in 5% CO_2_ for 48 h after which they were treated with either **1**–**12** (20 μM), or the positive control verapamil (100 μM), and incubated for 15 min. Cells were then treated with calcein AM (0.25 μM) and incubated for a further 30 min. Fluorescence of the calcein AM hydrolysis product calcein was quantified (POLARstar Omega multimode plate reader) and displayed in Figure 2 as the mean value ± SEM of two independent experiments performed in duplicate (* *p* < 0.05), with the dashed line corresponding to the PBS negative control.

The Calcein AM accumulation assay (96-well plate format, Section 3.3) [19] was used as the primary screen to assess P-gp inhibitory properties of **1**–**12**, with a compound designated as an inhibitor if a 20 μM treatment increased calcein fluorescence ≥30% of that exhibited by a 100 μM treatment with the positive control verapamil. While the majority of metabolites tested did not exhibit inhibitory activity against P-gp, **11** displayed a moderate response (85% of the positive control) (Figure 2), an observation confirmed by cell flow cytometry (Section 2.3 and Section 2.4) and MDR reversal (Section 2.5) assays.

**Figure 3:**
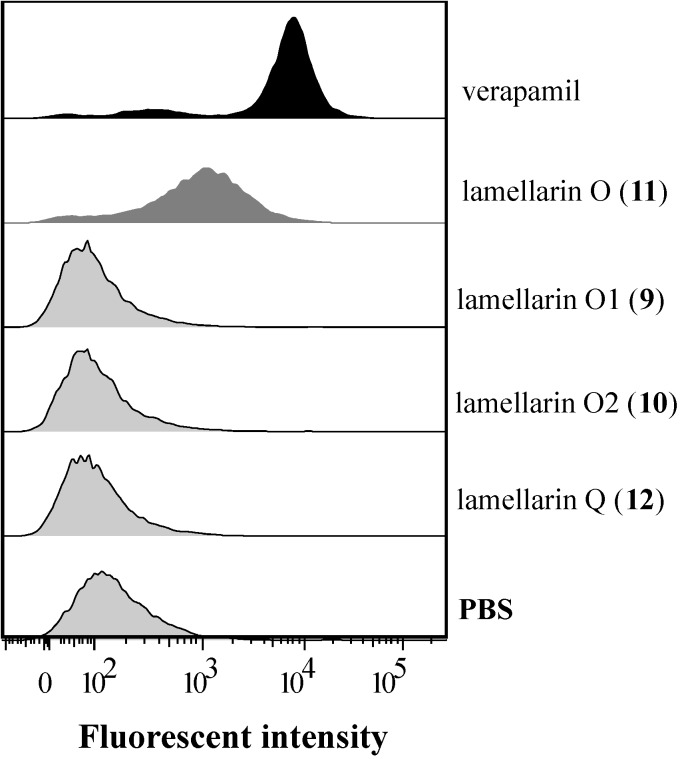
Effect of **9**–**12** on accumulation of calcein AM in SW620 Ad300 cells using flow cytometry. SW620 Ad300 cells were incubated with calcein AM (0.25 μM) with or without **9**–**12** (20 μM), or the positive control verapamil (20 μM), after which cells were washed twice with ice-cold PBS and the intracellular calcein fluorescence levels quantified using flow cytometry (Section 3.4). The histogram results (Y-axis represents cell counts with the maximum as 400) for **9**–**12** and verapamil are illustrated, with data for other metabolites reported in Supplementary Table S2.

### 2.3. Lamellarin O (**11**) as a P-gp Inhibitor in SW620 Ad300 Cells (Calcein AM by Cell Flow Cytometry)

Cell flow cytometry is an established methodology that when coupled with the Calcein AM assay provides a reliable and accurate means to quantify P-gp inhibitors [20]. Calcein AM assay coupled with cell flow cytometry (Section 3.4) yielded results that were in accord with those detailed above (96-well plate format, Section 2.2) and confirmed that **11** (20 μM) exhibited a moderate (5.1-fold) inhibitory effect on the accumulation of calcein AM from SW620 Ad300 cells (Figure 3 and Supplementary Table S2), with the remaining co-metabolites exhibiting no inhibitory activity (<1.0 fold).

**Figure 4:**
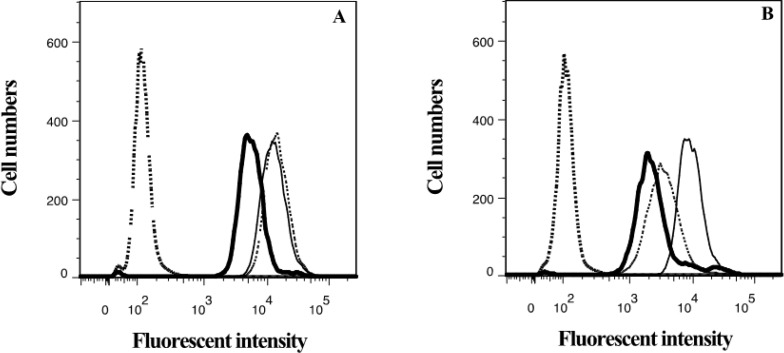
Effect of lamellarin O (**11**) and verapamil on accumulation (**A**) and efflux (**B**) of Hoechst 33342 in SW620 Ad300 using flow cytometry. SW620 Ad300 cells were incubated with or without Hoechst 33342 (60 μM), in the absence or presence of **11** (20 μM), or verapamil (20 μM), for 30 min. After incubation cells were washed twice with ice-cold PBS, and intracellular calcein fluorescent intensity measured by flow cytometry (accumulation, Figure A). In an efflux phase assay SW620 Ad300 cells were further incubated in Hoechst 33342-free medium, with or without **11** (20 μM), or verapamil (20 μM) for 1 h. After which cells were washed as indicated above for the accumulation phase, and intracellular calcein fluorescent intensity measured by flow cytometry (Figure B). The accumulation and efflux phase increase in intracellular fluorescence mediated by **11** (thin dashed line) and verapamil (thin solid line), compared with the negative controls of PBS with Hoechst 33342 (heavy solid line), were 2.5-fold and 1.4-fold, and 2.3-fold and 3.8-fold respectively. Heavy dashed line illustrates baseline intracellular fluorescence of SW620 Ad300 cells without Hoechst 33342.

### 2.4. Lamellarin O (**11**) as a P-gp Inhibitor in SW620 Ad300 Cells (Hoechst 33342 Accumulation/Efflux)

To further validate the P-gp inhibitory properties of **11** we used cell flow cytometry to quantify the accumulation and efflux of Hoechst 33342 from P-gp over-expressing SW620 Ad300 cells (Section 3.4). In the accumulation phase treatment with **11** (20 μM) increased intracellular Hoechst 33342 fluorescence levels (2.5-fold) comparable to that achieved by the positive control verapamil (2.3-fold) (Figure 4A), whereas in the efflux phase treatment with **11** resulted in an increase in intracellular Hoechst 33342 fluorescence levels (1.4-fold) below than that achieved by verapamil (3.8-fold increase (Figure 4B). These results are consistent with the Calcein AM accumulation (96-well plate format, Section 2.2) and cell flow cytometry (Section 2.3) results detailed above, and confirm that **11** is a moderate inhibitor of P-gp mediated efflux.

### 2.5. Lamellarin O (**11**) Reverses P-gp Mediated Doxorubicin Resistance in SW620 Ad300 Cells

P-gp over-expressing colon cancer SW620 Ad300 cells treated with 5.0, 10 and 15 μM aliquots of **11** (Table 1) exhibited 1.5-, 3.1- and 4.8-fold increases in sensitivity towards doxorubicin, a well-known cancer chemotherapeutic and P-gp substrate. By contrast, treatment with 2.5 μM verapamil resulted in a 8.8-fold increase in sensitivity. These results are consistent with calcein AM accumulation (Section 2.2 and Section 2.3), and Hoechst 33342 accumulation/efflux (Section 2.4) assays, establishing **11** as a moderate P-gp inhibitor capable of reversing P-gp mediated doxorubicin resistance in SW620 Ad300 cells.

**Table 1:** Effect of lamellarin O (**11**) on P-gp mediated doxorubicin resistance in SW620 Ad300 cells ^a^.

Treatment	IC_50_^a^ (nM)	DMF ^b^
doxorubicin	4440 ± 210	
+ lamellarin O (**11**) 5.0 μM	2910 ± 350	1.5
+ lamellarin O (**11**) 10 μM	1430 ± 160	3.1
+ lamellarin O (**11**) 15 μM	930 ± 120	4.8
+ verapamil 2.5 μM	500 ± 230	8.8

^a^ Cell survival was determined by MTT assay (Section 3.5). Data are mean values ± SEM of at least three independent experiments performed in duplicate; ^b^ DMF: Dose-modifying factor was the ratio of IC_50_ value of doxorubicin against SW620 Ad300 without an inhibitor to IC_50_ value of doxorubicin against SW620 Ad300 with an inhibitor.

### 2.6. Lamellarin O (**11**) Reverses BCRP Mediated Efflux in NCI-H460/MX20 Cells

The flow cytometry based mitoxantrone efflux assay (Section 3.4) [19] was used to screen the BCRP inhibitory properties of **1**–**12** against the mitoxantrone resistant human lung cancer NCI-H460/MX20 cells, with a compound designated an inhibitor if a 20 μM treatment increased intracellular mitoxantrone fluorescence ≥50% of that exhibited by a 10 μM treatment of the positive control FTC [21,22]. While the majority of metabolites tested did not exhibit inhibitory activity against BCRP, the increase in intracellular mitoxantrone exhibited by **11** (3.05-fold, 94.5%) was comparable to that of FTC (3.17-fold, 100%), indicating that **11** was a promising BCRP inhibitor (Table 2).

**Table 2:** Effect of **1**–**12** on BCRP mediated efflux in NCI-H460/MX20 cells.

Treatment	FAR ^a^	% FTC ^b^
lamellarin O (**11**)	3.05	94.5
ianthellidone A (**1**)	0.97	<1.00
ianthellidone B (**2**)	1.07	3.13
ianthellidone C (**3**)	1.12	5.60
ianthellidone D (**4**)	1.15	6.66
ianthellidone E (**5**)	1.14	6.20
ianthellidone F (**6**)	1.21	9.79
ianthellidone G (**7**)	1.09	4.06
ianthellidone H (**8**)	1.13	6.06
lamellarin O1 (**9**)	1.23	10.6
lamellarin O2 (**10**)	1.11	5.13
lamellarin Q (**12**)	1.41	18.6
PBS	1.00	0.00
FTC 10 μM	3.17	100

^a^ FAR (fluorescence arbitrary ratio) = mitoxantrone fluorescence intensity (geometric mean) in the presence of metabolites from *Ianthella* sp. (CMB-01245) at 20 μM/mitoxantrone fluorescence intensity (geometric mean) in the presence of PBS, expressed as a ratio. Positive control is FTC at 10 μM which FAR = 3.17; ^b^ activities for inhibition on mitoxantrone efflux were normalized to 10 μM FTC response according to the following equation: % FTC = (RFU _test sample_ − RFU _negative_)/(RFU _FTC_ − RFU _negative_) × 100%; RFU _test sample_ is relative intracellular mitoxantrone fluorescent unit (geometric mean) in the presence of compound (20 μM), RFU _FTC_ is relative intracellular mitoxantrone fluorescent unit (geometric mean) in the presence of 10 μM FTC (positive control) and RFU _negative_ is relative intracellular mitoxantrone fluorescent unit (geometric mean) in the absence of sample (PBS only). A sample was determined to inhibit BCRP when % maximum was >50% [21,22].

### 2.7. Lamellarin O (**11**) Reverses Efflux of [^3^H]-Mitoxantrone in NCI-H460/MX20 Cells

The BCRP inhibitory activity exhibited by **11** was further verified by evaluating its effect on the intracellular accumulation and efflux of [^3^H]-mitoxantrone. Briefly, in accumulation phase, parental cells (NCI-H460) and BCRP over-expressing daughter cells (NCI-H460/MX20) were cultured with [^3^H]-mitoxantrone (0.10 μM) in the presence or absence of FTC (5.0 μM), or **11** (10 or 30 μM). After 1 h incubation, the intracellular [^3^H]-mitoxantrone was measured using a liquid scintillation analyzer, quantifying the ability of **11** to increase the uptake of the BCRP substrate [^3^H]-mitoxantrone. In the efflux phase, cells from the accumulation phase were washed to remove extracellular [^3^H]-mitoxantrone and incubated with additional FTC (5.0 μM), or **11** (10 or 30 μM) in media without [^3^H]-mitoxantrone. By measuring intracellular and media levels of [^3^H]-mitoxantrone it was possible to quantify the effect of FTC and **11** on BCRP mediated efflux of a [^3^H]-mitoxantrone.

**Figure 5:**
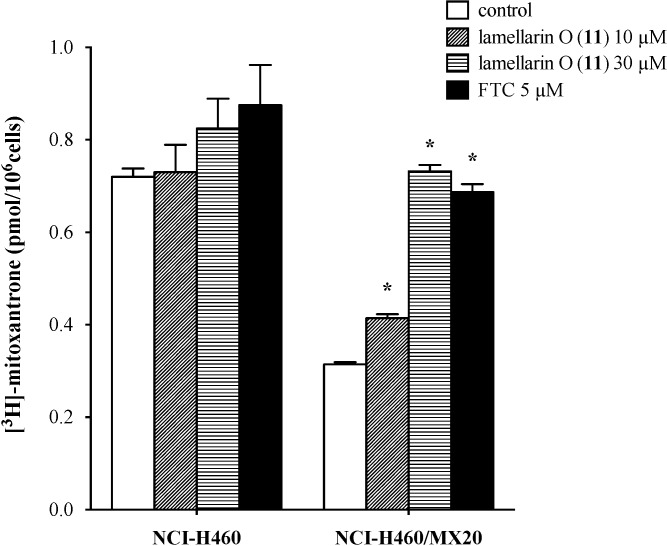
Effect of **11** on the accumulation of [^3^H]-mitoxantrone. The accumulation of [^3^H]-mitoxantrone in parental NCI-H460 cells and in BCRP over-expressing NCI-H460/MX20 cells was measured (Section 3.6). Columns are the mean of triplicate determinations. Error bars represent the SD; * *p* < 0.05 *vs.* the control group. Experiments were performed in three independent times, and a representative experiment is shown.

In the accumulation phase neither **11** (10 or 30 μM) nor FTC (5.0 μM) significantly changed intracellular [^3^H]-mitoxantrone levels in NCI-H460 cells, however, by contrast **11** increased [^3^H]-mitoxantrone accumulation in NCI-H460/MX20 cells (1.3- and 2.3-fold by 10 and 30 μM, respectively) comparable to the increase observed for FTC (2.2-fold) (Figure 5).

**Figure 6:**
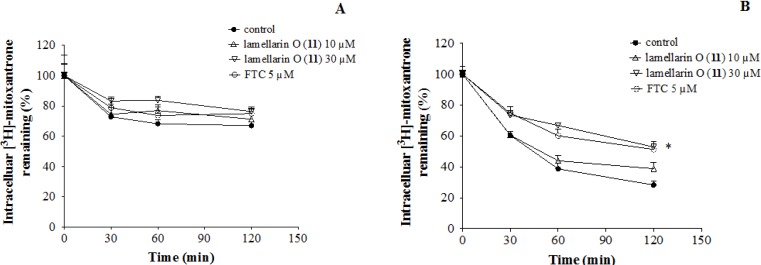
Effect of **11** on the efflux of [^3^H]-mitoxantrone in (**A**) NCI-H460 cells and (**B**) NCI-H460/MX20 cells. A time course *vs.* % of intracellular [^3^H]-mitoxantrone remaining was plotted; * *p* < 0.05 *vs.* control group. Error bars represent the SD. Experiments were carried out in triplicate.

In the efflux phase the intracellular level of [^3^H]-mitoxantrone in BCRP over-expressing NCI-H460/MX20 cells (Figure 6B) was significantly less than that in parental cells (Figure 6A), however, when treated with **11** (10 and 30 μM) NCI-H460/MX20 cells displayed a concentration dependent increase in intracellular [^3^H]-mitoxantrone levels, comparable with treatment with FTC (5.0 μM). Neither **11** nor FTC had a significant effect on the intracellular levels of [^3^H]-mitoxantrone in parental cells (Figure 6). This study indicates that **11** is a promising BCRP inhibitor, with the potential to increase uptake and decrease efflux of cancer chemotherapeutics in BCRP over-expressing cells.

### 2.8. Effect of Lamellarin O (**11**) on BCRP Expression in NCI-H460/MX20 Cells

**Figure 7:**
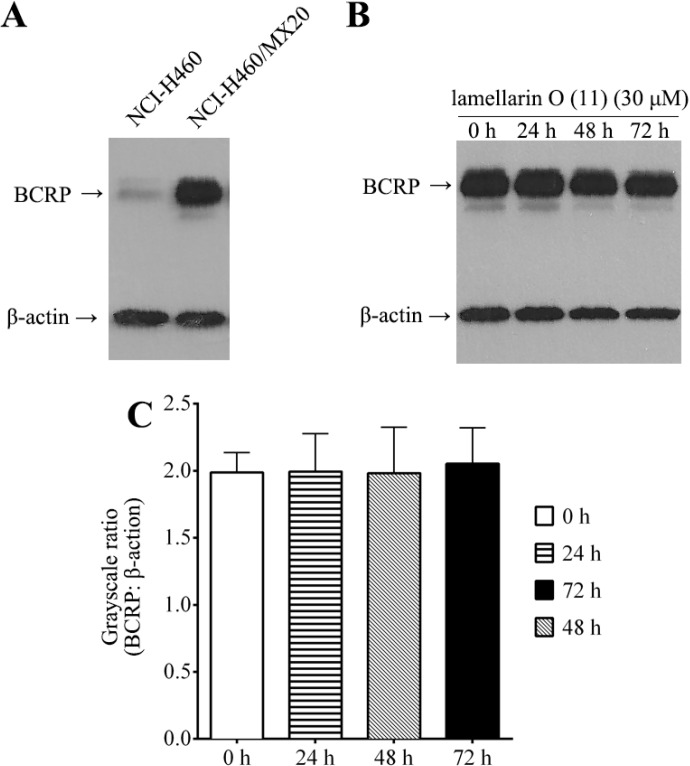
Western blotting analysis of BCRP and effect of **11** on BCRP expression in NCI-H460/MX20 cells. (**A**) Expression of BCRP in NCI-H460 and NCI- H460/MX20 cells; (**B**) Effect of **11** (30 μM) on expression level of BCRP in NCI-H460/MX20 cells (0, 24, 48 and 72 h); (**C**) Quantitative analysis on relative expression levels of ABCG2. Columns are grayscale ratios of BCRP over β-actin. Error bars represent SD. Equal amounts of total cell lysate were used for each Western blotting (Section 3.7). Representative results are shown, with similar results obtained in two other trials.

In addition to possibly acting as a small molecule inhibitor of BCRP functionality, an alternative mechanism by which **11** may decrease BCRP mediated cellular efflux of [^3^H]-mitoxantrone is via decreasing cellular levels of BCRP. In order to test this alternative mechanism we incubated NCI-H460 MX20 cancer cells in the absence (0 h) and in the presence of 30 μM **11** (24, 48 and 72 h), and determined BCRP expression using Western blotting analysis. As illustrated (Figure 7A) BCRP was expressed significantly in NCI-H460/MX20 cells, but slightly in NCI-H460 cells. The BCRP levels in NCI-H460/MX20 cells were not significantly changed after 24, 48 or 78 h (Figure 7B,C), confirming that **11** does not alter BCRP expression.

### 2.9. Structure Activity Relationship (SAR) Studies on Lamellarin O (**11**)

**Figure 8:**
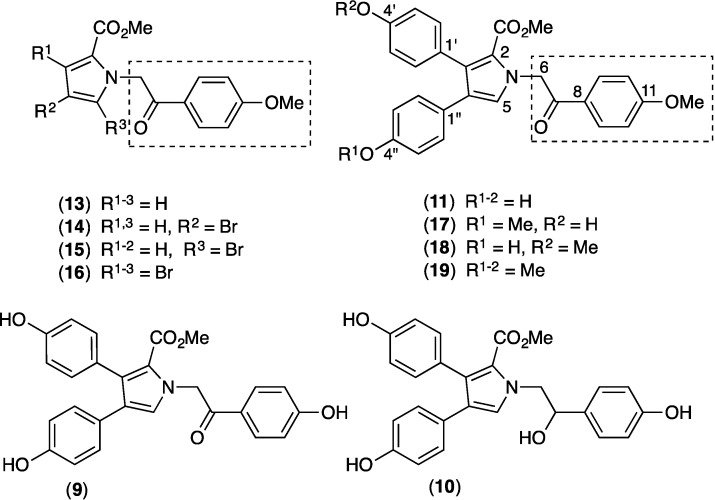
SAR studies on lamellarin O (**11**) with the methoxy-acetophenone moiety highlighted.

The results as outlined above established **11** as a significantly more potent BCRP inhibitor than the biosynthetically related co-metabolites **1**–**10** and **12**, indicative of a structure-activity relationship (SAR) in which the methoxy-acetophenone moiety (highlighted in Figure 8) may be a critical determinant. In light of this we reasoned that further SAR studies, including an evaluation of methylated analogues incorporating the core methoxy-acetophenone moiety, would be highly informative. To support SAR studies we sourced the synthetic analogues **13**–**16** from an in-house compound library, and prepared the mono and dimethyl analogues **17**–**19**. All these analogues incorporate the methoxy-acetophenone moiety unique to **11**, but absent from the closely related and BCRP inactive **9**–**10**.

**Figure 9:**
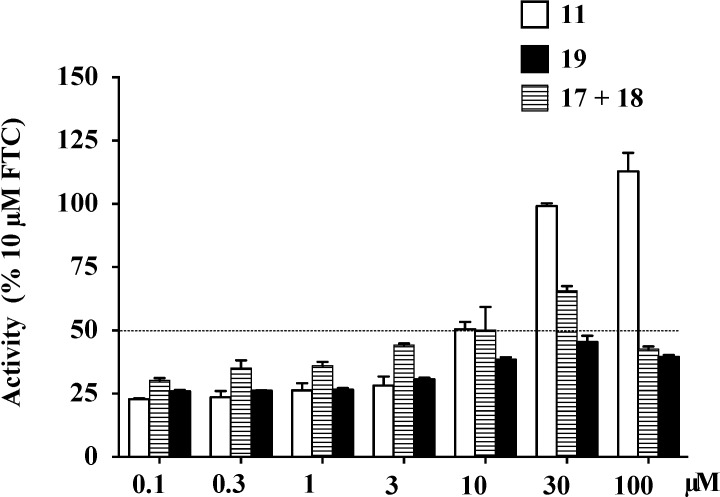
Effect of **11** and its methylated derivatives **17**–**19** on the BCRP mediated efflux of mitoxantrone in BCRP over-expressing NCI-H460/MX20 cells. NCI-H460/MX20 cells were incubated with mitoxantrone in the absence or presence of **11**, mix of **17**+**18**, or **19** (0.1 to 100 μM), or the positive control FTC (10 μM), after which the cells were washed twice with ice-cold PBS, and incubated without or with test compounds for a further 1 h in mitoxantrone free medium. Mitoxantrone fluorescence was detected with a 638-nm argon laser and a 660/20 nm band-pass filter. Data were collected by cell flow cytometry according (Section 3.4) and normalized to that obtained with 10 μM FTC (signal set as 100%). Data are means ± SEM of at least three independent experiments performed in duplicate.

Evaluation of **13**–**16** using the flow cytometry based mitoxantrone efflux assay (Section 3.4) showed that none of these compounds inhibited BCRP (<10% of 10 μM FTC, Supplementary Table S3). The BCRP inhibitory properties of **17**+**18** (mixture) and **19** were also evaluated over a range of concentrations (0.1 to 100 μM), and compared to **11**, using the flow cytometry based mitoxantrone efflux assay (Table 3, Figure 9). This analysis confirmed the IC_50_ and maximal responses (%FTC) for **11 ** (4.7 μM and 113%) were far superior to those for **17**+**18** (20 μM and 66%) and **19** (100 μM and 46%). 

**Table 3:** The effect of lamellarin O (**11**) and methylated derivatives **17**–**19** on BCRP mediated efflux of mitoxantrone in BCRP over-expressing NCI-H460/MX20 cells.

	IC_50_ (μM) ^a^	Maximal Activity (% of FTC)
**11**	4.7 ± 0.6	112.9
**17+18**	20.2 ± 2.1	65.7
**19**	>100	45.4

^a^ IC_50_ values were calculated from dose-response data using Prism analysis.

While limited, these SAR results nevertheless confirm that replacing the phenyl substituents pendant to the pyrrole moiety in **11** with bromo groups (e.g., **13**–**16**), or mono/di methylation of phenolic moieties (e.g., **17**–**19**), leads to a significant reduction in BCRP inhibitory activity. From this we conclude that while the methoxy-acetophenone moiety in **11** appears essential to delivering BCRP inhibitory activity, as seen by the loss of activity in **9**–**10**, by itself this moiety is not sufficient to deliver a BCRP inhibitory effect.

### 2.10. Docking Analysis of Lamellarin O (**11**) and 19 with Human ABCG2 Homology Models

**Figure 10:**
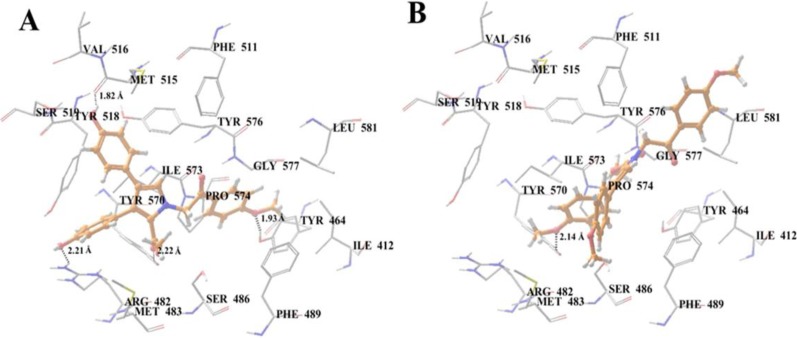
XP-Glide predicted binding mode of lamellarin O (**11**) with homology modeled ABCG2. (**A**) Binding mode of **11** within the drug binding site-1 of human ABCG2. Important residues are depicted as sticks with the atoms colored as carbon–gray, hydrogen–white, nitrogen–blue, oxygen–red, sulfur–yellow whereas **11** is shown as ball and stick model with the same color scheme as above except carbon atoms are represented in orange. Dotted black lines indicate proposed hydrogen bonds; (**B**) Compound **19** within the drug binding site-1 of human ABCG2.

To support SAR analyses we compared docking studies on the BCRP inhibitor **11**, with those on the BCRP inactive analogue **19**. In these studies, docking of **11** into the large drug-binding cavity of human ABCG2 revealed hydrogen bonding between the two phenolic residues, and Arg482 (-O···HN-Arg482, 2.2 Å) and Met515 (-OH···OC-Met515, 1.8 Å), respectively (Figure 10A). Similarly, the ester and methoxy-acetophenone moieties were seen to engage in hydrogen bonding with Tyr570 (-COO···HO-Tyr570, 2.2 Å) and Tyr464 (-O···HO-Tyr464, 1.9 Å), respectively, while **11** was further stabilized in the hydrophobic pocket by several hydrophobic and aromatic interactions. Importantly, hydrogen bonding to the phenol residues provides an insight into the likely mechanism behind the loss of BCRP inhibitory activity observed in the dimethylated analogue **19** as shown in Figure 10B.

### 2.11. The Effect of Lamellarin O (**11**) on the MRP1 Mediated Efflux of Calcein in 2008/MRP1 Cells

**Figure 11:**
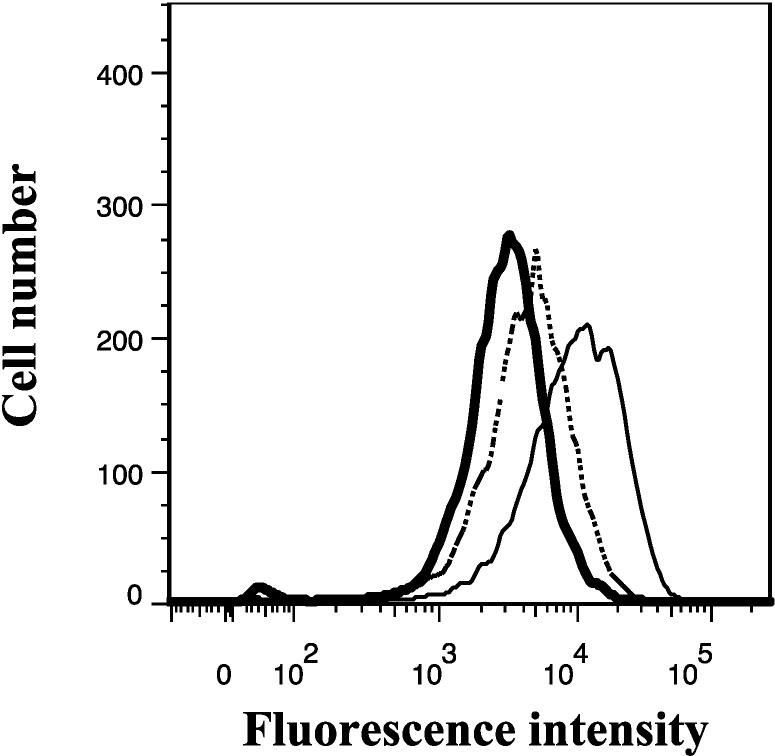
Effect of lamellarin O (**11**) on the efflux of MRP1 fluorescent substrate calcein in MRP1 over-expressing 2008/MRP1 cells. Cells were incubated for 30 min with calcein AM in the absence (heavy solid line) or presence (dashed line) of **11** (20 μM), or the positive control MK571 (50 μM) (thin solid light). Subsequently, cells were washed twice with ice-cold PBS and incubated without or with compounds for 1 h in calcein AM-free medium. Intracellular fluorescence of calcein was detected with a 488 nm argon laser and a 530/30 nm band pass filter. Data were analyzed by flow cytometry according (Section 3.4). Representative results from at least two independent experiments were shown.

Inspired by its P-gp and BCRP inhibitory properties, we used the Calcein AM assay supported by cell flow cytometry (Section 3.3 and Section 3.4) to examine the effect of **11** on the MRP1 mediated efflux of calcein AM. Treatment of 2008/MRP1 cells with 20 μM of **11** revealed only a modest increase in intracellular calcein fluorescence (1.4-fold), below than that exhibited by treatment with 50 μM of the positive control MK571 (3.2-fold), confirming that **11** is a weak inhibitor of MRP1 (Figure 11).

## 3. Experimental Section

### 3.1. Chemical Materials

Doxorubicin was a gift from BioAustralis Fine Chemicals (Smithfield, NSW, Australia). MK571 was purchased from Cayman Chemical Company (Ann Arbor, MI, USA). Fumitremorgin C (FTC), originally prepared by Thomas McCloud (Developmental Therapeutics Program, Natural Products Extraction Laboratory, NIH, Bethesda, MD, USA), was provided by Susan E. Bates and Robert W. Robey. [^3^H]-mitoxantrone (2.5 Ci/mmol) was supplied from Moravek Biochemicals Inc. (Brea, CA, USA). Monoclonal antibody BXP-21 (against ABCG2) was purchased from Signet Laboratories Inc. (Dedham, MA, USA). Calcein AM and Hoechst 33342 were purchased from Enzo Life Inc. (Plymouth Meeting, PA, USA). Dimethylsulfoxide (DMSO) was acquired from Chem-Supply Pty. Ltd. (Gillman, SA, Australia). G418 was supplied from Invitrogen (Carlsbad, CA, USA). Pheophorbide A (PhA) was purchased from Frontier Scientific, Inc. (Logan, UT, USA). 3-(4,5-dimethylthiazol-2-yl)-2,5-diphenyltetrazolium bromide (MTT), mitoxantrone, verapamil, propidium iodide, and other chemicals were obtained from Sigma-Aldrich Pty. Ltd. (Castle Hill, NSW, Australia).

The marine natural products **1**–**12**, reported in 2012 by Zhang *et al.* [23] from a southern Australian marine sponge, *Ianthella* sp. (CMB-01245), and the structurally related synthetic compounds **13**–**16** were available from one of the authors (Robert J. Capon). The analogues **17**–**19** were prepared from the natural product **11** as described below. All compounds (**1**–**19**) were subjected to HPLC-DAD-MS and ^1^H NMR analysis prior to detailed biological experiments.

### 3.2. Cell Lines and Cell Culture

Three human cancer cell lines (SW620, NCI-H460 and 2008) and their ABC transporter over-expressing counterparts (SW620 Ad300, NCI-H460/MX20 and 2008/MRP1) were examined in this study. The drug-resistant P-gp over-expressing daughter cell line SW620 Ad300 was established by stepwise selection of the parental human colon cancer cell line SW620 in increasing concentrations of doxorubicin, and was maintained in RPMI1640 (Invitrogen, Carlsbad, CA, USA) containing doxorubicin (300 ng/mL). The drug-resistant BCRP over-expressing daughter cell line NCI-H460/MX20 was established by stepwise selection of the parental human non-small cell lung cancer cell NCI-H460 in increasing concentrations of mitoxantrone, and was maintained in RPMI1640 containing mitoxantrone (20 nM). These four cell lines were kindly provided by Susan E. Bates and Robert W. Robey of the National Cancer Institute, Bethesda, MD, USA [24]. In order to confirm their drug resistance characteristics, the drug-selected MDR cell lines (SW620 Ad300 and NCI-H460/MX20 were transferred to drug-free medium until two weeks before assays. The human ovarian carcinoma cell line 2008 and its ABCC1 transduced sub-line 2008/MRP1 [25] were generously provided by Johan Allen (The University of Sydney) with permission from Piet Borst (the National Cancer Institute, Amsterdam, The Netherlands). All cell lines were grown in flasks in a humidified incubator at 37 °C with 5% CO_2_ with RPMI 1640, which was supplemented with 10% fetal bovine serum, l-glutamine (2 mM), penicillin (100 unit/mL) and streptomycin (100 μg/mL) (Invitrogen, Carlsbad, CA, USA).

### 3.3. Calcein AM Accumulation Assay (96-Well Plate Format)

The Calcein AM accumulation assay (96-well plate format) was modified based on the original method [26]. The non-fluorescent reagent calcein AM can diffuse into the cellular cytoplasm and yield the fluorescent dye calcein after hydrolysis by intracellular esterases. As calcein AM is a P-gp substrate, in the presence of functioning P-gp it is largely effluxed prior to hydrolysis, resulting in low levels of intracellular fluorescence. A P-gp inhibitor can block the efflux of calein AM, which then undergoes hydrolysis to calcein which is not a P-gp substrate, leading to higher levels of intracellular fluorescence. Briefly, after harvesting with trypsin, 5 × 10^4^ SW620 Ad300 cells (in 100 μL) were seeded into a well of 96-well flat clear-bottom black-wall microtitre plates (AP, Falcon, 353219, BD, Franklin Lakes, NJ, USA). Following 48 h incubation at 37 °C with 5% CO_2_, wells were treated with an aliquot (25 μL) of test compounds (20 μM), or verapamil (100 μM as a positive control), or 1% DMSO in PBS (v/v, negative control), for 15 min at 37 °C with 5% CO_2_. An aliquot (25 μL) of calcein AM (0.25 μM) was then added to each well, except for background control wells, which were treated with a 25 μL PBS. Plates were incubated at 37 °C with 5% CO_2_ for a further 30 min, after which intracellular calcein fluorescent intensity (arbitrary unit) was measured by a POLARstar Omega plate reader (BMG LABTECH, Offenburg, Germany) at 490 nm (excitation wavelength) and 510 nm (emission wavelength). Data (fluorescence unit, FU) were normalized to verapamil treated cells on the same microtitre plate, and reported as % of 100 μM verapamil activity according to:

% maximum = (FU_sample_ − FU_negative_)/(FU_verapamil_ − FU_negative_) × 100 (1)

where FU_sample_ is fluorescence in the presence of test compounds (20 μM), FU_verapamil_ is fluorescence in the presence of verapamil (100 μM) and FU_negative_ is fluorescence in the absence of sample (negative control). A sample with P-gp inhibitory activity was determined when % maximum was >30%. All experiments were performed in duplicate.

### 3.4. Flow Cytometry Assays

Flow cytometry based assays were used to measure the ability of natural or synthetic compounds to inhibit P-gp, BCRP or MRP1 mediated accumulation and/or efflux of fluorescent reagents [21]. Briefly, SW620 Ad300 cells (P-gp over-expressing), NCI-H460/MX20 cells (BCRP over-expressing), or 2008/MRP1 cells (MRP1 over-expressing), were harvested with trypsin and re-suspended in phenol red-free RPMI 1640 (with 10% FBS) to give a final concentration of 50 × 10^4^ cells/mL. Cells were then pre-incubated with 20 μM of test compounds, or 20 μM of verapamil (positive control for P-gp), or 10 μM FTC (positive control for BCRP) or 50 μM MK571 (positive control for MRP1) for 15 min at 37 °C in 5% CO_2_. For the comparison of BCRP inhibitory activity of lamellarin O (**11**) and its permethylated derivatives (mix of **17**+**18**, or **19**), NCI-H460/MX20 cells were treated with compounds at a series of concentration (0.1, 0.3, 1, 3, 10, 30 and 100 μM). Subsequently, cells were incubated with reagents (0.25 μM calcein AM or 60 μM Hoechst 33342 for P-gp, 20 μM of mitoxantrone for BCRP, and 0.25 μM calcein AM for MRP1) for 30 min followed by washing twice with cold medium. For the calcein AM accumulation assays, cells were directly analyzed by flow cytometry. For the efflux assays, cells treated with Hoechst 33342 (P-gp), mitoxantrone (BCRP) or calcein AM (MRP1) were incubated for 1 h in fluorescence reagent free medium with or without compounds, or corresponding positive controls (see above), after which cells were washed twice with cold medium.

Intracellular fluorescence intensity was measured on a BD FACSCanto™ II flow cytometer (Becton Dickinson, San Jose, CA, USA) and data were analyzed through FlowJo (Tree Star, Inc., Ashland, OR, USA). Calcein was detected with a 480 nm laser and a 530/30 nm band-pass filter, whereas Hoechst 33342 was detected using a 405 nm laser and a 450/50 nm band-pass filter. Mitoxantrone fluorescence was detected with a 638 nm argon laser and a 660/20 nm band-pass filter. At least 10,000 events were collected for all of the flow cytometry experiment. By increasing threshold value on forward scatter *versus* side scatter, debris was eliminated. Dead cells were excluded based on propidium iodide staining. Data are means ± SEM of at least three independent experiments performed in duplicate.

### 3.5. MTT Cytotoxicity Assay

The MTT assay was modified based on that previously described [27], using adherent cell lines SW620 and NCI-H460, and their P-gp over-expressing daughter cell line SW620 Ad300 and BCRP over-expressing daughter cell line NCI-H460/MX20, respectively. Briefly, cells were harvested with trypsin and added into 96-well microtitre assay plates at 2000 cells/well, followed by 18 h incubation at 37 °C with 5% CO_2_. Lamellarin O (**11**) was dissolved in 5% DMSO in PBS (*v/v*) and aliquots (20 μL) tested over a series of concentrations. Control wells were treated with 5% aqueous DMSO. When the assay was used for P-gp MDR reversal activity, SW620 Ad300 cells were co-incubated with 5, 10 or 15 μM aliquots of lamellarin O (**11**), or 2.5 μM verapamil, together with doxorubicin at a series of concentrations. 68 h later, 20 μL MTT (4 mg/mL in PBS) was dispensed into each well (final concentration 0.40 mg/mL), and plates were incubated for a further 4 h at 37 °C with 5% CO_2_. Finally medium was removed and precipitated formazan crystals were dissolved in 100% DMSO. The absorbance of each well was measured at 580 nm with a PowerWave XS Microplate Reader from Bio-Tek Instruments Inc. (Vinooski, VT, USA).

IC_50_ values were calculated using Prism 5.0 (GraphPad Software Inc., La Jolla, CA, USA), as the concentration of analyte required for 50% inhibition of cancer cell growth (compared to negative controls). All experiments were performed in duplicate.

### 3.6. Drug Accumulation and Efflux Assay

The effect of lamellarin O (**11**) on the intracellular accumulation of mitoxantrone in BCRP over-expressing cells was determined by measuring the intracellular accumulation of [^3^H]-mitoxantrone in NCI-H460 and NCI-H460/MX20 cells. Cells were trypsinized and three aliquots (5 × 10^6^ cells) from each cell line were resuspended in medium and pre-incubated for 1 h at 37 °C with or without 10 or 30 μM lamellarin O (**11**), or 5 μM FTC. To measure drug accumulation, cells were subsequently incubated for 2 h at 37 °C with 5% CO_2_ with 0.1 μM [^3^H]-mitoxantrone in RPMI 1640 medium, in the presence or absence of 10, 30 μM lamellarin O (**11**), or 5 μM FTC. The resulting cells were pelleted at 4 °C, washed three times with 10 mL ice-cold PBS, placed in lysis buffer, and radioactivity measured using a Packard TRI-CARB 1900CA liquid scintillation analyzer from Packard Instrument Company Inc. (Downers Grove, IL, USA) as previously described [28].

In the efflux study cells were incubated with 0.1 μM [^3^H]-mitoxantrone as the same in the accumulation study. After washing three times with cold PBS, the suspended cells were incubated at 37 °C in fresh medium in the presence or absence of 10 or 30 μM lamellarin O (**11**), or 5 μM FTC. After 0, 30, 60, and 120 min aliquots of cells were removed and immediately washed three times with 10 mL of ice-cold PBS. The resulting cells were pelleted and processed for measurement of radioactivity (as indicated above).

### 3.7. Preparation of Total Cell Lysates and Western Blotting

Cells were treated with 30 μM lamellarin O (**11**) for 0, 24, 48 and 72 h, after which cell lysates were prepared as previously described [28] and stored at −80 °C until the gel electrophoresis was run. Protein concentrations were determined by bicinchonic acid (BCA™) based protein assay (Thermo Scientific, Rockford, IL, USA). Equal amounts of total cell lysates (20 μg protein) were resolved by SDS-PAGE and electrophoretically transferred onto PVDF membranes. Following 1 h incubation in a blocking solution in TBST buffer at room temperature, the membranes were immunoblotted for 2 h at room temperature with primary monoclonal antibodies against actin, at a 1:400 dilution, or ABCG2, at a 1:100 dilution. The membranes were then further incubated for 1 h at room temperature with HRP-conjugated secondary antibody (1:2000 dilution). Finally the protein-antibody complex was detected by enhanced chemiluminescence detection system (Amersham, NJ, USA). In addition, levels of protein expression were quantified by ImageJ Software (NIH, Bethesda, MD, USA).

### 3.8. Syntheses of Methylated Lamellarin O Derivatives (**17**, **18** and **19**)

A solution of **11** (7.7 mg, 0.017 mmol) in acetone (2 mL) was treated with a 3-fold excess of MeI (7.2 mg, 0.051 mmol), and K_2_CO_3_ (14.0 mg, 0.10 mmol), and was stirred at ambient temperature overnight. After filtration the eluent was concentrated *in vacuo* and the residue purified by semi-preparative HPLC (Agilent Zorbax Rx-C_8_, 5 μm, 9.4 × 250 mm column, 15 min gradient elution at 3.5 mL/min from 90% H_2_O/MeCN to 100% MeCN with an isocratic 0.01% TFA/H_2_O modifier) to afford 4′-methyl-lamellarin O (**18**); *t*_R_ 13.52 min, 3.9 mg, 50%), 4″-methyl-lamellarin O (**17**); *t*_R_ 13.7 min, 2.4 mg, 30%) and 4′,4″-dimethyl-lamellarin O (**19**); *t*_R_ 15.4 min, 0.6 mg, 8.0%).

A solution of **11** (3 mg, 0.0066 mmol) in acetone (2 mL) was treated with a 10-fold excess of MeI (9.3 mg, 0.066 mmol), and K_2_CO_3_ (9.1 mg, 0.066 mmol), and was stirred at ambient temperature overnight. Workup and fractionation as detailed above yielded 4′,4″-dimethyl-lamellarin O (**19**) (*t*_R_ 15.4 min, 2.4 mg, 75%).

4′-methyl-lamellarin O (**18**): ^1^H NMR (600 MHz, CDCl_3_): δ_H_ = 8.03 (d, *J* = 8.7 Hz, 2H), 7.15 (d, *J* = 8.7 Hz, 2H), 7.00 (d, *J* = 8.7 Hz, 2H), 6.95 (d, *J* = 8.7 Hz, 2H), 6.92 (s, 1H), 6.82 (d, *J* = 8.7 Hz, 2H), 6.64 (d, *J* = 8.7 Hz, 2H), 5.74 (s, 2H), 3.90 (s, 3H), 3.82 (s, 3H), 3.47 (s, 3H); ^13^C NMR (150 MHz, CDCl_3_): δ_C_ = 192.1, 164.3, 162.7, 158.5, 154.0, 132.0, 131.3, 130.5, 129.8, 128.1, 128.1, 127.4, 127.3, 124.9, 120.0, 115.2, 114.3, 113.0, 55.7, 55.7, 55.3, 51; UV (MeOH) λ_max_ (log ε) 277 (4.48) nm; HRESI(−)MS *m/z* 494.1574 [M + Na]^+^ (calcd for C_28_H_25_NO_6_Na^+^, 494.1574).

4″-methyl-lamellarin O (**17**): ^1^H NMR (600 MHz, CDCl_3_): δ_H_ = 8.03 (d, *J* = 8.7 Hz, 2H), 7.10 (d, *J* = 8.7 Hz, 2H), 7.00 (d, *J* = 8.7 Hz, 2H), 7.00 (d, *J* = 8.7 Hz, 2H), 6.93 (s, 1H), 6.77 (d, *J* = 8.7 Hz, 2H), 6.72 (d, *J* = 8.7 Hz, 2H), 5.74 (s, 2H), 3.90 (s, 3H), 3.75 (s, 3H), 3.46 (s, 3H); ^13^C NMR (150 MHz, CDCl_3_): δ_C_ = 192.1, 164.2, 162.5, 158.0, 154.5, 132.2, 131.3, 130.5, 129.6, 128.2, 128.1, 127.3, 127.1, 124.9, 120.0, 114.6, 114.3, 113.7, 55.7, 55.7, 55.3, 51; UV (MeOH) λ_max_ (log ε) 277 (4.46) nm; HRESI(−)MS *m/z* 494.1574 [M + Na]^+^ (calcd for C_28_H_25_NO_6_Na^+^, 494.1574).

4′,4″-dimethyl-lamellarin O (**19**): ^1^H NMR (600 MHz, CDCl_3_): δ_H_ = 8.03 (d, *J* = 8.7 Hz, 2H), 7.16 (d, *J* = 8.7 Hz, 2H), 7.00 (d, *J* = 8.7 Hz, 2H), 7.00 (d, *J* = 8.7 Hz, 2H), 6.93 (s, 1H), 6.82 (d, *J* = 8.7 Hz, 2H), 6.72 (d, *J* = 8.7 Hz, 2H), 5.74 (s, 2H), 3.90 (s, 3H), 3.82 (s, 3H), 3.75 (s, 3H), 3.47 (s, 3H); ^13^C NMR (150 MHz, CDCl_3_): δ_C_ = 192.0, 164.2, 162.5, 158.4, 158.0, 132.0, 131.3, 130.5, 129.6, 128.1, 128.1, 127.4, 127.1, 124.9, 120.0, 114.3, 113.7, 113.0, 55.7, 55.7, 55.3, 55.3, 51.0; UV (MeOH) λ_max_ (log ε) 277 (4.68) nm; HRESI(−)MS *m/z* 508.1732 [M + Na]^+^ (calcd for C_29_H_27_NO_6_Na^+^, 508.1731).

### 3.9. Molecular Modeling

Lamellarin O (**11**) and 4′,4″-dimethyl-lamellarin O (**19**) were prepared as ligands for docking simulations into human ABCG2 following the same protocol as previously described [29]. All grids of ABCG2 were prepared and generated as per our pervious protocol [30]. Grid-1 generated using Arg482 had the highest docking score; therefore, docking discussion was based on binding model of **11** and **19** at this site. Glide v6.0 (Schrödinger, LLC, New York, NY, USA, 2013) docking tool was followed with the default functions. Top scoring conformation was used for graphical analysis. All computations were carried out on a Dell Precision 490n dual processor with Linux OS (Ubuntu 12.04 LTS, Canonical Group Limited, London, UK).

### 3.10. Statistical Analysis

The statistical significance was defined as *p* < 0.05 by two-tailed Student’s *t*-test. All experiments were performed at least three times in duplicate.

## 4. Conclusions

In this study we assessed the P-gp, BCRP or MRP1 inhibitory properties of a family of marine alkaloids, ianthellidone A–F (**1**–**9**) and lamellarins O1 (**9**), O2 (**10**), O (**11**) and R (**12**), isolated from a southern Australian marine sponge *Ianthella* sp. (CMB-01245), leading to the discovery of **11** as a potent and selective BCRP inhibitor. The P-gp, MRP1 and BCRP inhibitory properties of **11** were detected, measured and quantified by calcein AM accumulation and cell flow cytometry based (accumulation or efflux) assays against human colon cancer (SW620 and SW620 Ad300), ovarian carcinoma (2008 and 2008/MRP1) and non-small cell lung (NCI-H460 and NCI-460/MX20) cells, respectively, with further validation of P-gp inhibitory properties measured in Hoechst 33342 accumulation and efflux, and doxorubicin drug resistance reversal assays, and of BCRP inhibitory properties in [^3^H]-mitoxantrone drug accumulation and efflux assays. Collectively these studies demonstrated that, whereas **11** exhibited weak to moderate inhibitory activity against P-gp and MRP1, it was a selective and potent inhibitor of BCRP (IC_50_ 4.7 ± 0.6 μM). Further studies demonstrated that **11** did not alter (reduce) the expression of BCRP in NCI-460/MX20 cells, suggesting that it was acting (as predicted) as a small molecule inhibitor of BCRP function. An SAR analysis on the BCRP inhibitory properties of **11**, drawing on data for the co-metabolites **1**–**10** and **12**, and the synthetic analogues **12**–**19**, and supported by *in silico* docking studies on **11** and **19**, identified structural elements that were key to the inhibitory pharmacophore, including the methoxy-acetophenone, carboxylic ester and phenolic residues. Our discovery and characterization of **11** as a selective inhibitor of BCRP will hopefully prompt and inform future SAR investigations into this pharmacophore, to explore and advance its potential to deliver a clinically useful BCRP inhibitor therapeutic.

## Supplementary Files

Supplementary File 1 Supplementary Information (PDF, 966 KB)
