# Supplementary material for: Lamellarin O, a Pyrrole Alkaloid from an Australian Marine Sponge, Ianthella sp., Reverses BCRP Mediated Drug Resistance in Cancer Cells
Source: Mar Drugs. 2014 Jun 27;12(7):3818–37. doi: 10.3390/md12073818 (PMC4113800; doi:10.3390/md12073818)
Supplement: Supplementary File 1 — Supplementary Information (PDF, 966 KB) [file marinedrugs-12-03818-s001.pdf]

Supplementary Information

Table S1. Cytotoxicity of **1**–**12** against parental SW620 and P-gp over-expressing SW620 Ad300 cells [a].

Compounds	IC_{50} (μM) [b]		FR [c]
ianthellidone A (**1**)	>30.0	>30.0	-
ianthellidone B (**2**)	>30.0	>30.0	-
ianthellidone C (**3**)	>30.0	>30.0	-
ianthellidone D (**4**)	>30.0	>30.0	-
ianthellidone E (**5**)	>30.0	>30.0	-
ianthellidone G (**7**)	>30.0	>30.0	-
ianthellidone H (**8**)	>30.0	>30.0	-
ianthellidone F (**6**)	>30.0	>30.0	-
lamellarin O2 (**10**)	>30.0	>30.0	-
lamellarin O1 (**9**)	>30.0	>30.0	-
lamellarin Q (**12**)	>30.0	>30.0	-
lamellarin O (**11**)	22.0 ± 1.8	22.3 ± 2.3	1.00

[a]: Cell survival was determined by MTT assay as described in Section 3.5.; [b]: Data are means ± SEM of at least three independent experiments performed in duplicate; [c]: fold-resistance (FR) was determined by dividing the IC_{50} value of compound in P-gp over-expressing cancer cells by the IC_{50} value in sensitive cancer cells. "-": not calculated.

Table S2. Cell flow cytometry analysis of intracellular calcein fluorescence in the presence of **1**–**12** (**20** μM).

Compounds	FAR
lamellarin O (**11**)	5.12
ianthellidone A (**1**)	0.63
ianthellidone B (**2**)	0.52
ianthellidone C (**3**)	0.61
ianthellidone D (**4**)	0.60
ianthellidone E (**5**)	0.59
ianthellidone G (**7**)	0.67
ianthellidone H (**8**)	0.60
ianthellidone F (**6**)	0.61
lamellarin O2 (**10**)	0.60
lamellarin O1 (**9**)	0.61
lamellarin Q (**12**)	0.54
verapamil	27.6

FAR (fluorescence arbitrary ratio) = calcein fluorescence intensity (geometric mean) in the presence of compound at 20 μM divided by calcein fluorescence intensity (geometric mean) in the presence of PBS, expressed as a ratio. Positive control is verapamil at 20 μM which FAR = 27.6. Experiment detail is in Section 3.4.

Figure S1. Effect of lamellarin O (**11**) and verapamil (2.5 μM) on the sensitivity of P-gp over-expressing SW620 Ad300 cancer cells to doxorubicin. MTT (96 h) cytotoxicity assay was performed with a series of concentrations of doxorubicin on P-gp over-expressing SW620 Ad300 in the presence or absence of verapamil (2.5 μM) or **11** (5, 10 or 15 μM) (Section 3.5). The various concentrations of doxorubicin were indicated in the figure, the data points were the means ± SEM of duplicate determination. The above figure showed a representative result for verapamil and **11**. A summary of results was available in Table 1.

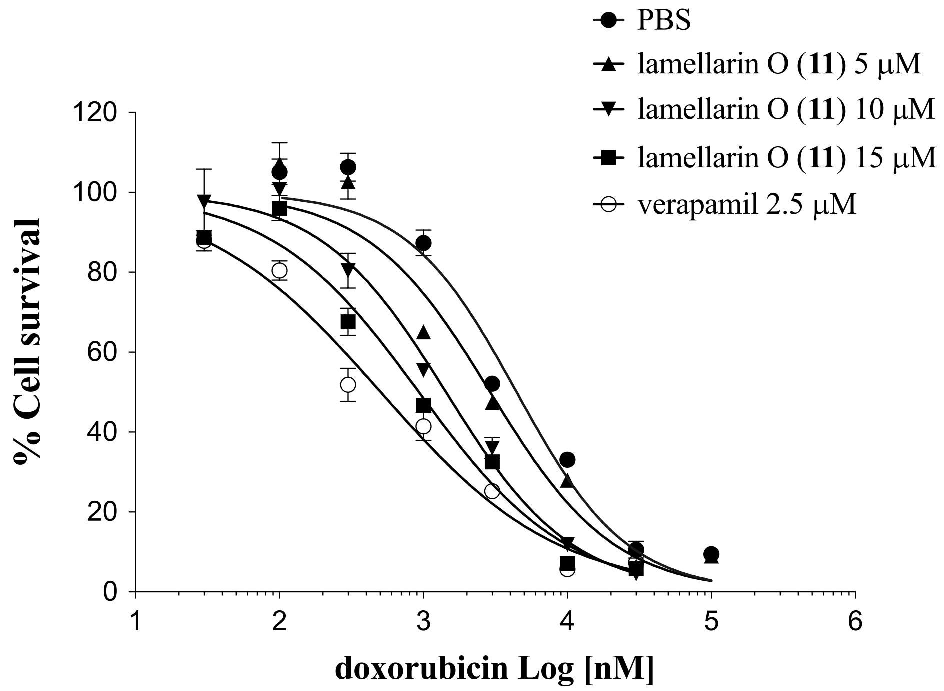

Table S3. Effect of **13**–**16** (20 μM) on BCRP mediated efflux of mitoxantrone in BCRP over-expressing NCI-H460/MX20 cells.

	FAR [a]	% FTC [b]
**13**	1.00	<1.00
**14**	1.03	<1.00
**15**	0.97	<1.00
**16**	1.20	5.45
PBS	1.00	0.00
FTC	3.17	100.00

[a] and [b] see details in Table 2.

Figure S2. ^{1}H NMR (600 MHz, $CDCl_3$) and UV-vis (MeOH) spectra of **18**.

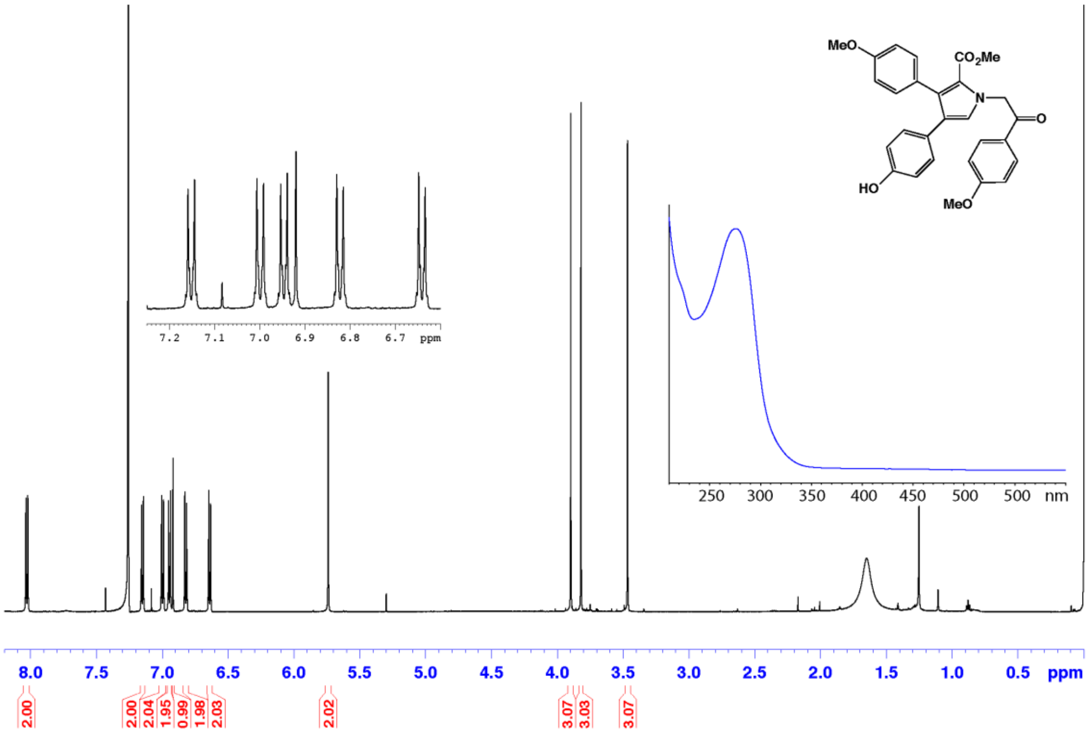

Figure S3. ^{13}C NMR (150 MHz, $CDCl_3$) spectrum of **18**.

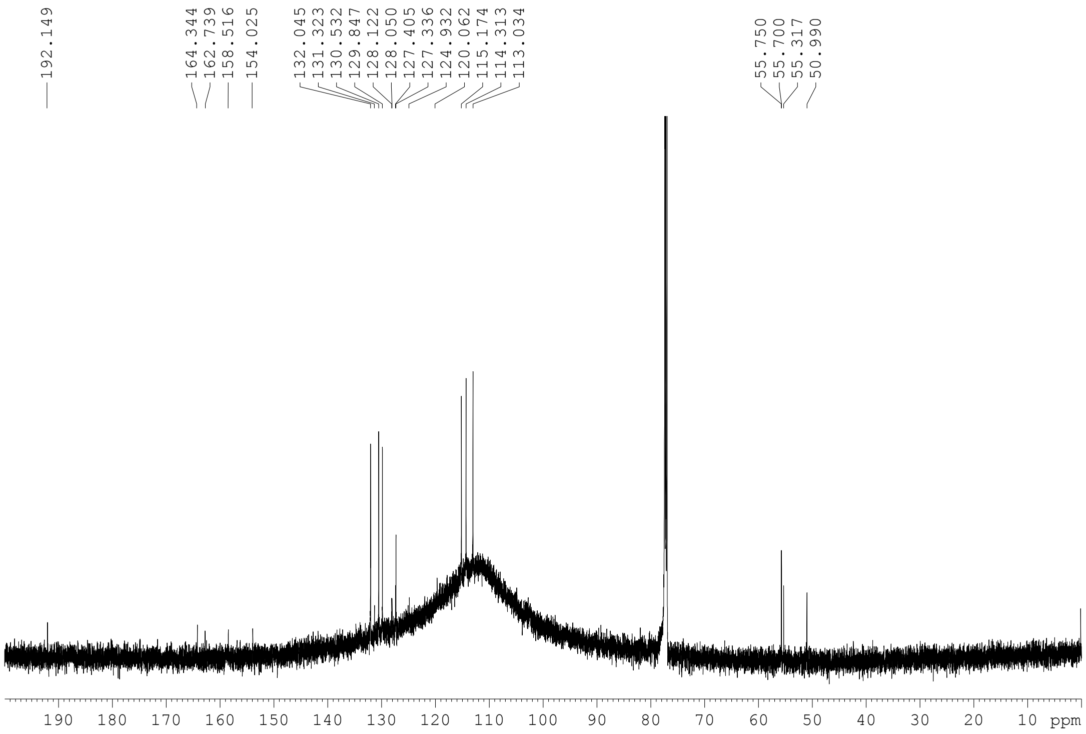

Figure S4. ^{1}H NMR (600 MHz, $CDCl_3$) and UV-vis (MeOH) spectra of **17**.

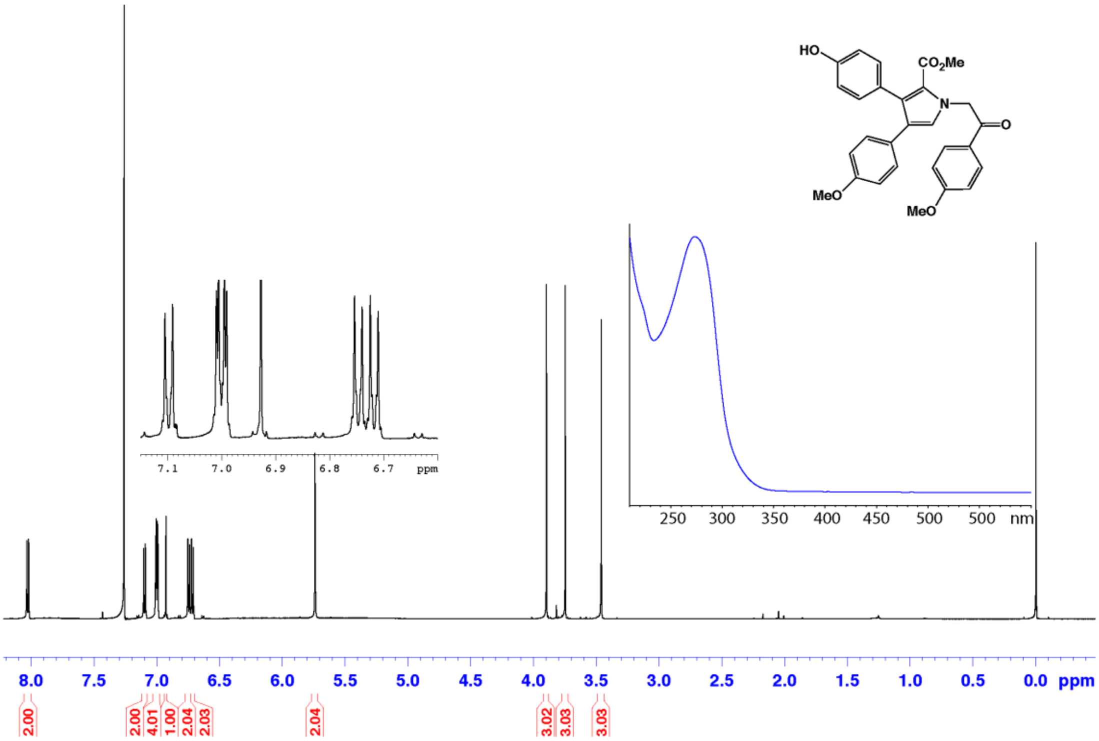

Figure S5. ^{13}C NMR (150 MHz, $CDCl_3$) spectrum of **17**.

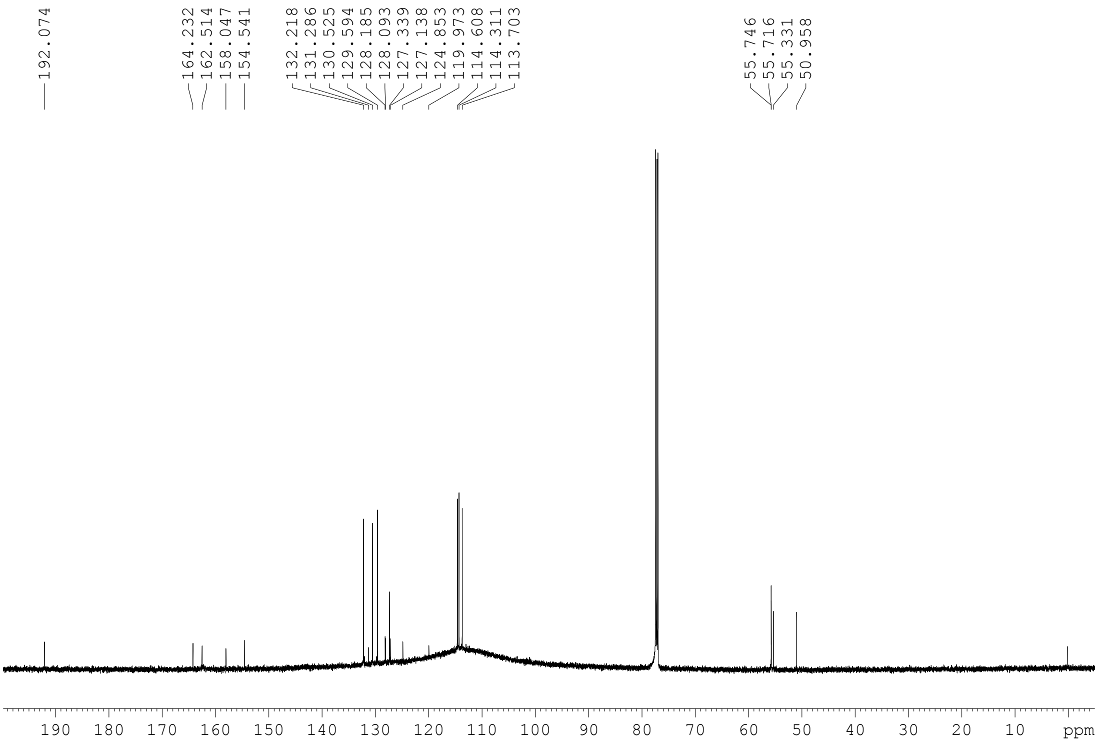

Figure S6. ^{1}H NMR (600 MHz, $CDCl_3$) and UV-vis (MeOH) spectra of **19**.

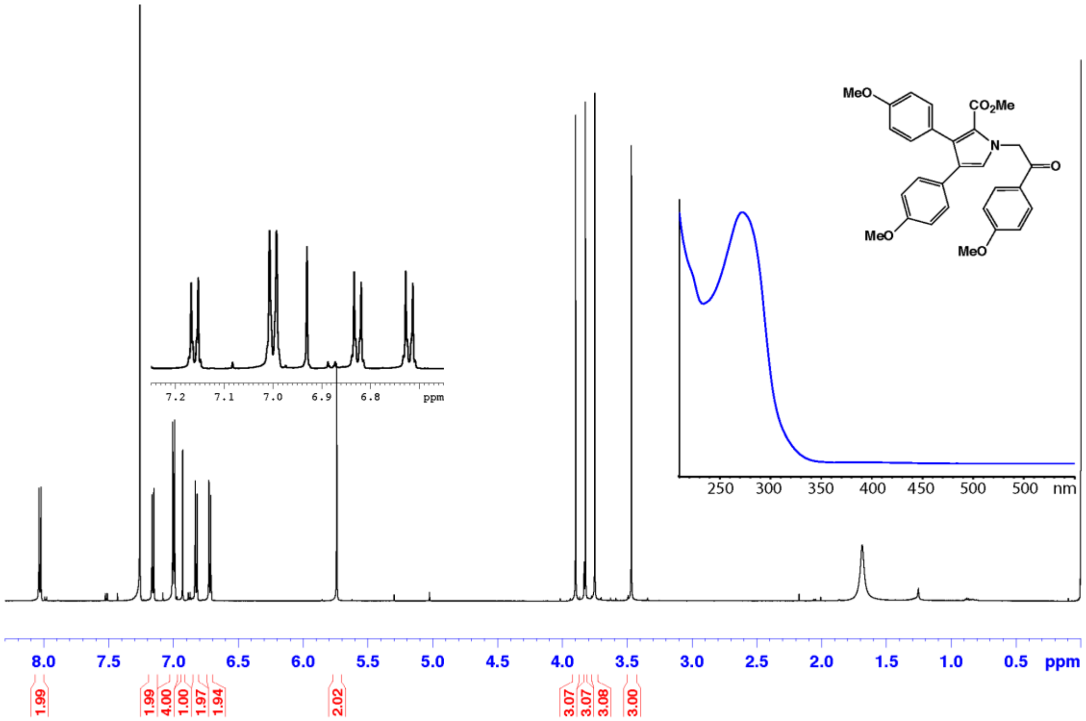

Figure S7. ^{13}C NMR (150 MHz, $CDCl_3$) spectrum of **19**.

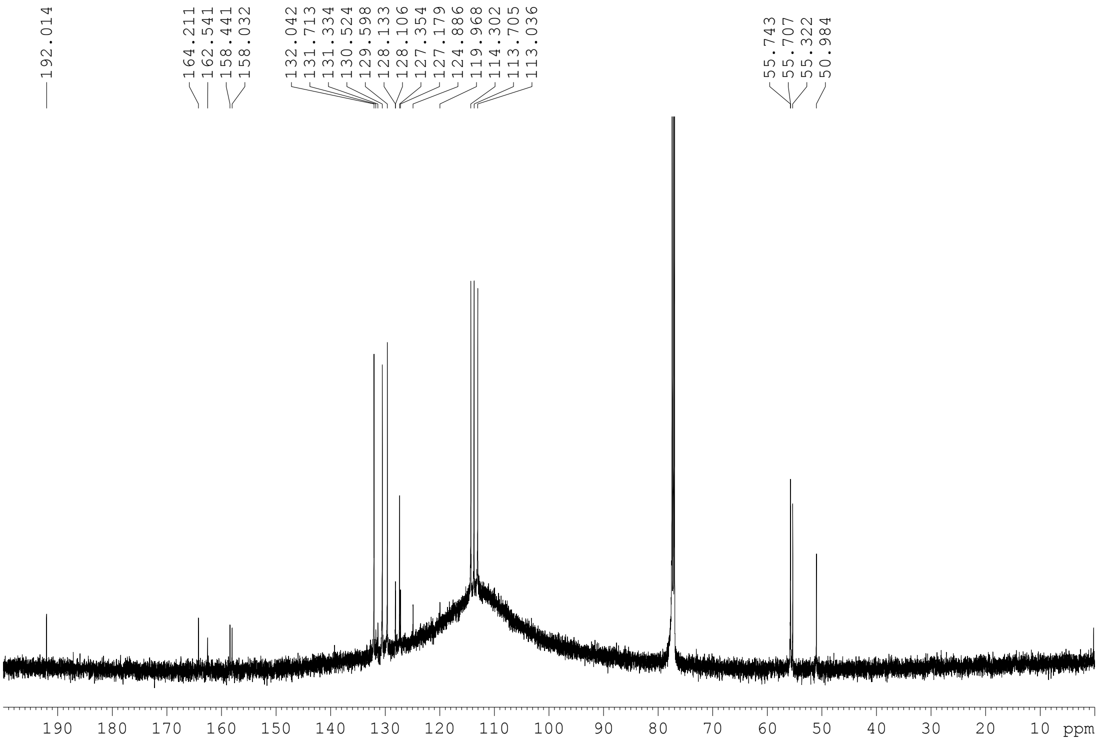

Figure S8. Selected HMBC (150 MHz, $CDCl_3$) correlations.

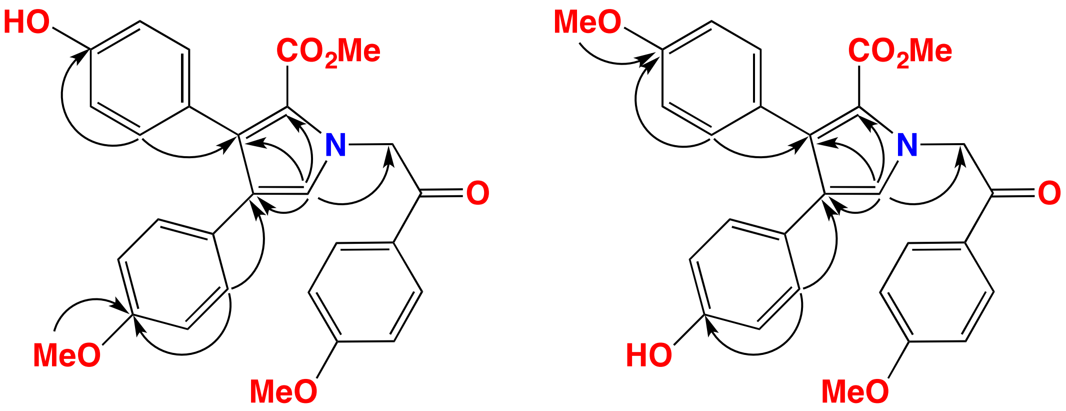

17 **18**

© 2014 by the authors; licensee MDPI, Basel, Switzerland. This article is an open access article distributed under the terms and conditions of the Creative Commons Attribution license (http://creativecommons.org/licenses/by/3.0/).
